# *In vitro* characterization of sonothrombolysis and echocontrast agents to treat ischemic stroke

**DOI:** 10.1038/s41598-019-46112-z

**Published:** 2019-07-09

**Authors:** Himanshu Shekhar, Robert T. Kleven, Tao Peng, Arunkumar Palaniappan, Kunal B. Karani, Shaoling Huang, David D. McPherson, Christy K. Holland

**Affiliations:** 1 Department of Internal Medicine, Division of Cardiovascular Health and Disease, University of Cincinnati, Cincinnati, OH USA; 2 Department of Biomedical Engineering, University of Cincinnati, Cincinnati, OH USA; 3 Department of Internal Medicine, Division of Cardiovascular Medicine, University of Texas Health Science Center-Houston, Houston, TX USA

**Keywords:** Biomedical engineering, Cardiac device therapy

## Abstract

The development of adjuvant techniques to improve thrombolytic efficacy is important for advancing ischemic stroke therapy. We characterized octafluoropropane and recombinant tissue plasminogen activator (rt-PA)-loaded echogenic liposomes (OFP t-ELIP) using differential interference and fluorescence microscopy, attenuation spectroscopy, and electrozone sensing. The loading of rt-PA in OFP t-ELIP was assessed using spectrophotometry. Further, it was tested whether the agent shields rt-PA against degradation by plasminogen activator inhibitor-1 (PAI-1). An *in vitro* system was used to assess whether ultrasound (US) combined with either Definity or OFP t-ELIP enhances rt-PA thrombolysis. Human whole blood clots were mounted in a flow system and visualized using an inverted microscope. The perfusate consisted of either (1) plasma alone, (2) rt-PA, (3) OFP t-ELIP, (4) rt-PA and US, (5) OFP t-ELIP and US, (6) Definity and US, or (7) rt-PA, Definity, and US (n = 16 clots per group). An intermittent US insonation scheme was employed (220 kHz frequency, and 0.44 MPa peak-to-peak pressures) for 30 min. Microscopic imaging revealed that OFP t-ELIP included a variety of structures such as liposomes (with and without gas) and lipid-shelled microbubbles. OFP t-ELIP preserved up to 76% of rt-PA activity in the presence of PAI-1, whereas only 24% activity was preserved for unencapsulated rt-PA. The use of US with rt-PA and Definity enhanced lytic efficacy (*p* < 0.05) relative to rt-PA alone. US combined with OFP t-ELIP enhanced lysis over OFP t-ELIP alone (*p* < 0.01). These results demonstrate that ultrasound combined with Definity or OFP t-ELIP can enhance the lytic activity relative to rt-PA or OFP t-ELIP alone, respectively.

## Introduction

Stroke causes 6.5 million deaths worldwide every year^1^ and remains the fifth leading cause of death in the United States^2^. Ischemic stroke, in which blood supply to the brain is blocked, accounts for up to 87% of all strokes^2^. Recombinant tissue-type plasminogen activator (rt-PA) is the only United States Food and Drug Administration (FDA) approved lytic agent for treating ischemic stroke^3,4^. Administration of rt-PA is limited to the first 4.5 hours after symptom onset in eligible patients to obtain therapeutic benefit with reduced risk of sequelae such as intracerebral hemorrhage^5,6^. The efficacy of rt-PA is affected by composition^7^, size^8^, and location^9,10^ of thrombi, and time of lytic administration after symptom onset^11^. Among individuals with large vessel occlusions treated with rt-PA, less than 35% experience favorable outcomes^12^. Recent randomized clinical trials have demonstrated the efficacy of mechanical thrombectomy performed within 6–8 h of stroke onset^13–16^. Subsequent studies showed that a subset of patients with large vessel occlusions could be treated with mechanical thrombectomy within 16–24 hours of symptom onset^17–19^. Although these advances have produced a paradigm shift in stroke therapy, mechanical thrombectomy is feasible only at specialized stroke centers^20^. Intravenous thrombolytic therapy is still the primary strategy for treating eligible patients in the majority of clinical settings. Furthermore, rt-PA is often administered before mechanical thrombectomy to prevent clot embolization^15^, or before transfer to a specialized stroke center^21^. Therefore, the development of adjuvant techniques to improve rt-PA efficacy remains important.

Ultrasound (US) exposure of echocontrast agents has been investigated as an adjuvant to enhance rt-PA thrombolysis in previous *in vitro*^22–26^, *ex vivo*^27^, and *in vivo* studies^28–30^. Our group has reported enhanced thrombolysis in the presence of 120-kHz US with echogenic liposomes^31^. These agents contain rt-PA and octafluoropropane gas-loaded vesicles (OFP t-ELIP). The exact location of the gas pockets in OFP t-ELIP has not yet been elucidated. However, gas pockets stabilized by a monolayer within the liposome, or within the bilayer shell are hypothesized to be responsible for the echogenicity of these vesicles^31^. We have also demonstrated 120-kHz US-enhanced thrombolysis in the presence of rt-PA and Definity (Lantheus Medical Imaging, North Billerica, MA), an FDA approved contrast agent^32,33^. Stable cavitation and acoustic radiation force were identified as the mechanisms for the lytic enhancement^22,32^. Although these results were encouraging, large microbubbles (50 ± 19 μm diameter) were observed after 120 kHz exposures of Definity *in vitro*^32^, which could pose an embolization risk^34^. Microbubbles smaller than the resonant size have been shown to coalesce rapidly due to secondary Bjerknes forces^35,36^. The diameter of a bubble resonant at 120-kHz frequency was calculated to be 50 μm^32^.

Reversible bioeffects have been reported in the nonhuman primate brain exposed to 220 kHz using a peak to peak acoustic pressure range of 300 to 600 kPa, in the presence of Definity^37^. The resonant microbubble diameter from insonation frequencies around 220 kHz (27 μm), has been shown to be free of embolic risk^34,38^. A 220-kHz frequency insonation has also been shown to penetrate the temporal bone adequately^39^. In this study, we assessed the morphology of echocontrast agents using differential interference contrast (DIC) and fluorescence microscopy to ascertain the location of OFP and rt-PA within these agents. Additionally, the size distribution, rt-PA loading, and acoustic attenuation coefficient of these agents was evaluated. Subsequently, we tested whether 220-kHz US in combination with cavitation nuclei – either OFP t-ELIP, or Definity enhances thrombolytic efficacy relative to treatment with rt-PA alone.

## Results

Spectrophotometric assessment revealed that undiluted OFP-tELIP contained 252 ± 9 μl of rt-PA per ml of solution in vial, which corresponds to a loading ratio of 84 ± 3%. The OFP-tELIP retained as much as 76 ± 2% of rt-PA enzymatic activity after exposure to PAI-1 (500 ng/ml) in solution. In contrast, free rt-PA retained only 24 ± 2% of its enzymatic activity when exposed to PAI-1 (500 ng/ml).

**Figure 1:**
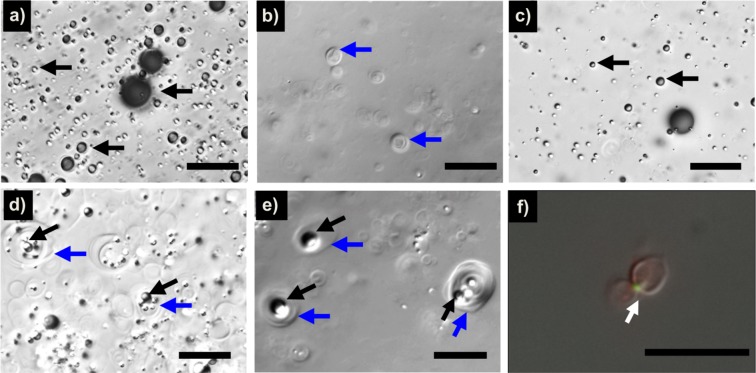
Differential interference contrast microscopy images of (**a**) Definity microbubbles (black arrows), (**b**) OFP t-ELIP reconstituted in undersaturated water (80 ± 2% dissolved gas saturation) showing only liposomes (blue arrows), (**c**) OFP t-ELIP reconstituted in air-saturated water (100 ± 2% dissolved gas saturation) showing microbubbles and a few liposomes near the top coverslip, (**d**) OFP t-ELIP showing a majority of liposomes which appear to entrap microbubble(s) near the bottom slide, (**e**) a close-up view of liposomes that entrap microbubbles, and (**f**) overlaid fluorescence and DIC images of FITC and rhodamine B stained OFP t-ELIP. Scale bar = 15 μm.

Figure 1(a) shows DIC images of Definity, which contains microbubbles with a single type of structure. A polydisperse population of microbubbles (black arrows) is visible – the majority of microbubbles are smaller than 5 µm in diameter, consistent with the previously reported size distribution of the agent^40,41^. The microbubbles appear dark or bright depending on the location within the focal depth of field. Figure 1(b) shows OFP t-ELIP reconstituted in undersaturated water (80 ± 2% dissolved gas saturation), which contained liposomes without gas loading These liposomes appear as faintly visible structures with a “relief-like” appearance (blue arrows). Figure 1(c,d) show OFP t-ELIP reconstituted in air-saturated water (100 ± 2% dissolved gas saturation) imaged by focusing near the top coverslip or the bottom slide, respectively. Near the top coverslip (Fig. 1(c)), buoyant microbubbles surrounded by lipid monolayer similar to Definity are visible. Although the dilution of microbubbles based on particle number density shown in Fig. 1(a,c) was similar, fewer microbubble-like particles appeared in the OFP t-ELIP sample due to their small percentage in the formulation. Near the bottom slide (Fig. 1(d)), OFP t-ELIP structures showing a majority of liposomes that appear to entrap microbubble(s) are visible. Another image with a magnified view of such OFP t-ELIP structures confirmed the presence of microbubbles that are entrapped within liposomes (Fig. 1(e)). In the Supplementary Video 1, these liposomes with entrapped microbubbles are seen moving within the microscopy field. Figure 1(f) shows a DIC image of OFP t-ELIP overlaid with fluorescent images of fluorescein isothiocyanate (FITC) tagged to rt-PA and rhodamine B. DIC microscopy images reveal the lipid bilayers of the liposomes. Two liposome structures that appear to be converging are observed. FITC (green, indicated by the arrow) appears to be co-localized with lipid (stained red) in a liposome. These images demonstrate the co-localization of rt-PA and lipid in the liposomes”.

**Figure 2:**
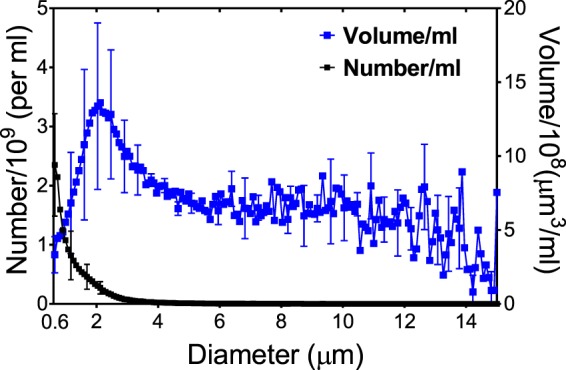
The number- (left ordinate) and volume-weighted (right ordinate) size-distributions of OFP t-ELIP (octafluropropane- and recombinant tissue plasminogen activator-loaded echogenic liposomes). The error bars represent the mean ± 1 standard deviation (n  =  3).

Figure 2 shows the number-weighted and volume-weighted size distributions of OFP t-ELIP. The mean number-weighted and volume-weighted diameters of OFP t-ELIP were 1.35 ± 0.02 μm and 8.23 ± 0.04 μm, respectively. More than 99.2% of the particles had a diameter less than 7 μm.

**Figure 3:**
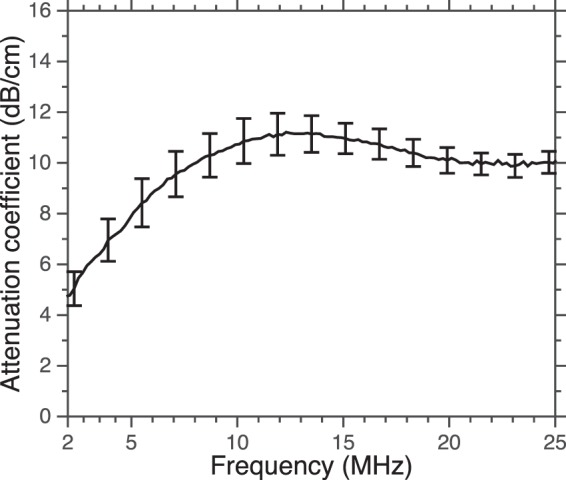
The attenuation coefficient of OFP t-ELIP (octafluropropane and recombinant tissue plasminogen activator-loaded echogenic liposomes) measured as a function of frequency (2–25 MHz). The error bars represent the mean ± 1 standard deviation (n = 3).

The attenuation spectrum of OFP t-ELIP is depicted in Fig. 3. The attenuation spectrum showed a broadband response. A peak in attenuation was observed at 12 MHz.

**Figure 4:**
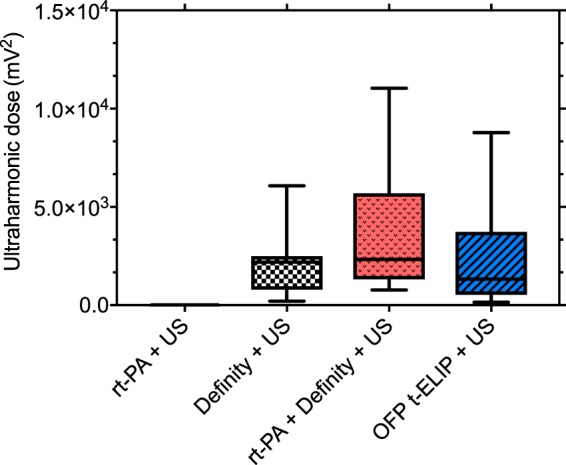
The ultraharmonic dose obtained with plasma alone, OFP t-ELIP, and Definity in response to intermittent ultrasound (n = 16 per group). The boxes represent the interquartile range, the horizontal lines within the boxes represent the median values, and the whiskers represent 1.5 times the interquartile range. For each group, n = 16 clots were tested. All groups were significant relative to rt-PA + US (*p* < 0.01). No difference was found between the ultraharmonic dose achieved from OFP t-ELIP and Definity (*p* > 0.05).

Figure 4 shows ultraharmonic cavitation dose measured for control and treatment arms in response to 220 kHz insonation. The administration of cavitation nuclei increased the stable cavitation significantly (*p* < 0.05) relative to the insonification of plasma only. However, no differences were observed in the cavitation dose measured from OFP t-ELIP and Definity (p > 0.05).

**Figure 5:**
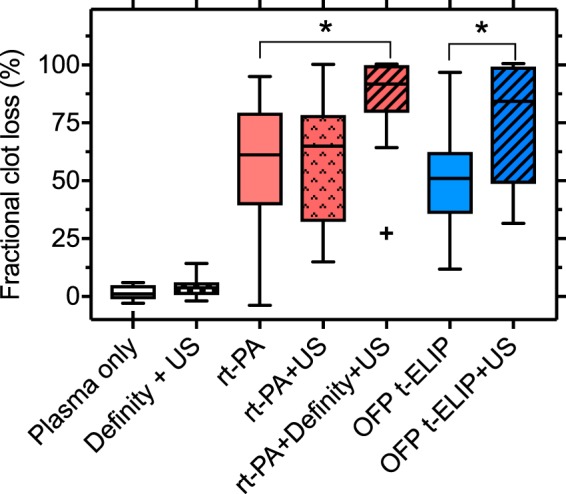
Fractional clot loss obtained with clots exposed to plasma alone, rt-PA, rt-PA + US, rt-PA + Definity + US, and rt-PA + Definity + US. For each group, n = 16 clots were tested. The error bars represent ±1 standard deviation. The ‘+’ sign denotes an outlier. Differences (*p* < 0.05) across treatments are denoted by (*).

Figure 5 shows the fractional clot loss (FCL)^31^ obtained for the control and treatment arms in the study. The adjuvant use of US with rt-PA and Definity produced an enhancement (*p* = 0.039) in FCL. However, FCL achieved with US combined with OFP t-ELIP was not different from that achieved with rt-PA alone (p > 0.05). Additionally, the combination of US and OFP t-ELIP produced significant enhancement in FCL over OFP t-ELIP alone (*p* = 0.001). No difference was observed in the FCL of US combined with rt-PA and Definity, or OFP t-ELIP (*p* > 0.05) without US exposure. Supplementary Video 2 shows a representative longitudinal cross section of a clot suspended by a suture inside a transparent glass capillary tube in the *in vitro* flow system, as it is being exposed to 220-kHz intermittent US and Definity. The pixel resolution of these images is 7.4 × 7.4 µm. No bubbles close to the resonant size were observed in this study, in contrast to the work of Bader *et al*.^32^ who used the same *in vitro* system with 120 kHz intermittent ultrasound and Definity.

**Figure 6:**
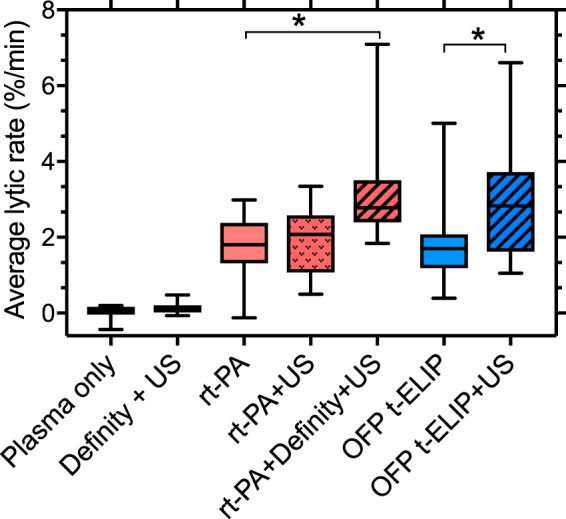
Average lytic rate obtained with clots exposed to plasma alone, rt-PA, rt-PA + US, rt-PA + Definity + US, and rt-PA + Definity + US. For each group, n = 16 clots were tested. The error bars represent ±1 standard deviation. Differences (*p* < 0.05) across treatments are denoted by (*).

Figure 6 shows the average lytic rate (ALR)^31^ obtained for the control and treatment arms in the study. The use of US with rt-PA and Definity produced enhanced ALR relative to rt-PA alone (*p* = 0.029). However, US combined with OFP t-ELIP did not improve ALR relative to rt-PA alone (*p* > 0.05). Combination of US and OFP t-ELIP produced a significant enhancement in ALR over OFP t-ELIP alone (*p* = 0.034). The ALR of US combined with rt-PA and Definity, and OFP t-ELIP with US exposure were not significantly different (*p* > 0.05).

## Discussion

We demonstrated that (a) rt-PA co-localized with lipid vesicles, some of which contained gas, (b) lipid loading can protect rt-PA against degradation by PAI-1, and (c) OFP t-ELIP and US can achieve equivalent thrombolytic efficacy relative to rt-PA, Definity and US.

In this study, we visualized the morphology of OFP t-ELIP and Definity using DIC and florescence microscopy. DIC microscopy is well-suited for visualizing transparent objects such as biological specimens. DIC generates contrast by evaluating the difference in the optical phase between two points in the sample plane^42^. These changes in the optical phase are related to changes in sample thickness and refractive index. In DIC images, the lipid bilayer surrounding the vesicles produces scant contrast relative to the aqueous media, making these structures faintly visible. However, the presence of gas bubbles is expected to produce a sharp contrast, making microbubbles visible prominently in DIC images. Kopecheck and colleagues visualized structures resembling microbubbles within ELIP by transmission electron microscopy^43^. However, sample preparation steps and the small field of view make it challenging to clearly interpret ELIP morphology using TEM. The DIC microscopy studies reported in the present manuscript provide further evidence that OFP t-ELIP contain drug-loaded liposomes that may or may not entrap a bubble, as well as bubble-like structures surrounded by a lipid monolayer. Microbubbles entrapped within liposomes or lipid monolayers could both contribute to US-mediated therapy. A small percentage of OFP t-ELIP particles had gas, which is consistent with our previous report^31^. Although undiluted OFP t-ELIP were visualized with DIC microscopy Fig. 1(a–e), for performing fluorescence imaging Fig. 1(f), we visualized a 100-fold diluted suspension of OFP t-ELIP, to be able to adjust the contrast for the red and green channels optimally for each particle of OFP t-ELIP before FITC was quenched by laser illumination. Because a majority of the particles did not contain gas, we imaged non-echogenic particles which were easier to locate in the field. In Fig. 1(e), two liposomes appear to be converging. Nonetheless, it is unlikely that convergence of liposomes affects the FITC signal, because it was acquired at a different wavelength than the DIC microscopy image. We oobserved the co-localization of lipids and rt-PA, which was consistent with the finding that loading echogenic liposomes with rt-PA preserves the enzymatic activity in the presence of PAI-1.

OFP t-ELIP showed a broadband response between 2 and 25 MHz. The frequency range of 2–25 MHz represents the 20-dB bandwidth of the attenuation spectroscopy system used in this study. Note that this range covers the conventional diagnostic frequency range and the lower end of the frequencies used in small animal imaging^41^. The majority of ultrasound contrast agents in clinical and preclinical use are micron-sized and demonstrate resonant behavior in the 2–25 MHz frequency range. Although the initial size of these agents was far from resonance at 220 kHz, the UCA used in the present study served nonetheless as cavitation nuclei for enhancing thrombolytic efficacy.

A fixed volume of blood (500 µl) was used to prepare the clots. The width of the clots was 410 ± 65 µm, which was smaller than the middle cerebral artery (2.4–4.6 mm)^44,45^. The length of the clots was nearly 20 mm. However, thrombolytic assessment was performed only within the field-of-view of the time-lapse microscopy system (i.e., 1.2 × 0.9 mm), although each clot was larger than these dimensions for ease of handling. The length and width of the clots varied slightly due to difference in the degree of retraction of clots from different donors. Histological analysis of thrombi retrieved from ischemic stroke patients via thrombectomy has revealed wide variability in composition, with clots being either predominantly erythrocytic, fibrin-rich, or a mixture of fibrin and erythrocytes^46–49^. Although clots used in the present study were prepared using venous blood, these clots were allowed to retract for 3 days. Clots prepared using this protocol are characterized by a dense fibrin network, presence or erythrocytes, and markedly increased resistance to rt-PA lysis than fresh venous clots^33,50^.

In a previous *in vitro* thrombolysis study reported by our group using 120-kHz US insonation, resonant bubbles (53 ± 19 µm in diameter), were observed intermittently^32^ and persisted for up to 100 s. Although Bader *et al*.^32^ reported dramatic thrombolysis in the presence of large bubbles, these bubbles could increase the risk of embolism. Despite the absence of large bubbles in the present study in contrast to the work of Bader and colleagues^32^ (compare Supplementary Video 2 to Supplementary Videos 1–3 reported by Bader *et al*.^32^), the FCL obtained in the present study was equivalent to our previous work^32^. Sustained ultraharmonic emissions were observed from all echocontrast agents used in this study. We have shown previously that perfluorocarbon-filled UCA produce higher ultraharmonic dose than air-filled agents^23,31^, likely due to the low solubility and diffusivity of OFP gas^51^.

A recent report^52^ showed that freeze-dried crystals of mannitol nucleated bubbles upon reconstitution in water, even without the presence of liposomes. Freeze-drying in the presence of mannitol, a cryoprotectant, is essential for producing echogenic liposomes^53^. It has been postulated that mannitol causes defects in the lipid bilayer during freezing, which traps gas. However, the studies performed by Kumar and colleagues^52^ demonstrate that mannitol nucleates bubbles (outgassing) by increasing the local concentration of sugar, which decreases the solubility of gas in the liquid. These investigators conjectured that bubbles nucleated both within the liposomes and in solution could account for the echogenicity of ELIP. The results of DIC microscopy reported in the present study are consistent with this proposed mechanism of echogenicity.

The particle concentration of OFP t-ELIP in this study was nearly 5-fold higher than Definity. The particle concentration was chosen based on the loading efficiency of OFP t-ELIP, to achieve a dose of 3.15 μg/mL of rt-PA. The concentration of Definity used in this study (2.4 × 10^7^ microbubbles/mL) and our previous work^32,33^ was higher than the clinically approved concentration for diagnostic imaging. However, previous studies in murine and canine models have shown that high concentrations of UCA (up to 250 times the clinically approved dose) may be well-tolerated^54^. There was no increase in lytic effect when clots were exposed to 220-kHz US and Definity without rt-PA. These results are consistent with previous work at 120 kHz that concluded that stable cavitation is not effective in lysing clots in the absence of rt-PA^32^. Insonation with 220-kHz US enhanced the lytic effect of OFP t-ELIP relative to that achieved with OFP t-ELIP alone. However, the lytic effect was not higher relative to rt-PA alone.

We modified our OFP t-ELIP formulation reported previously^31^ by replacing DPPC with DSPC as the main lipid constituent. It has been demonstrated that DSPC-shelled microbubbles are more stable against both passive and acoustically driven diffusion than DPPC-shelled microbubbles due to their longer phospholipid acyl chains^55–57^. The rt-PA to lipid ratio was also reduced by 25% in the present study. Consequently, the loading efficiency improved from 59 ± 12% in our previous study^31^ to 84 ± 3% in the present study.

The choice of US exposure parameters is critical for promoting thrombolysis without causing adverse bioeffects. We used 220-kHz insonation at a peak-to-peak pressure of 440 kPa. Thrombolysis at kilohertz frequencies and megahertz frequencies each have advantages and limitations. At kilohertz frequencies, acoustic attenuation through the skull is low^58,59^, which avoids temporal window insufficiency. Specifically, ultrasound frequencies lower than 500 kHz enable adequate penetration through the skull^58,59^. We have previously demonstrated that the broad beam associated with unfocused transducers can target clots lodged in the middle cerebral artery without image guidance^60^. Previous work reported in a primate model with 220-kHz US in the presence of UCA suggests that peak-to-peak pressures ranging from 300 to 600 kPa can cause reversible blood-brain barrier openings^37^. No other adverse effects were reported. In the context of ischemic stroke, blood brain barrier openings have been implicated with a moderate increase in the risk of hemorrhagic transformation^61^. An open blood brain barrier could potentially be exploited to administer neuroprotective drugs to promote survival of ischemic neurons^62^. However, sequelae related to low-frequency transcranial insonation were observed in the TRUMBI clinical trial^63^. Thirteen out of 14 patients treated with 300 kHz pulsed ultrasound and rt-PA suffered intracerebral hemorrhage^63^. *Post hoc* analysis of the acoustic parameters by Baron and colleagues showed that constructive interference between the transmitted US and the reflections from the contralateral bone could have increased *in situ* peak pressures substantially^64^. At kilohertz frequencies, constructive interference^60^ could have triggered hemorrhagic transformation in the presence of rt-PA and cavitation nuclei. The present study did not consider the effect of the presence of the skull on the therapy. Therefore, the potential for constructive interference and heating at the bone-tissue interface due to ultrasound absorption were not evaluated, which is a major limitation of this study. In the future, insonation strategies incorporating a short duty cycle, and chirp or random coding could help alleviate constructive interference in the brain^65^. Further, at megahertz frequencies, the likelihood of constructive interference within the skull is reduced, which improves the safety profile of therapy^60^. However, attenuation through the skill bone could be a major limiting factor for thrombolysis at MHz frequencies. Alternatively, sonothrombolysis with endovascular probes could also be considered for thrombolysis at MHz frequencies to avoid temporal bone insufficiency.

In this study, Definity combined with rt-PA significantly enhanced lysis relative to the use of rt-PA alone. However, the lytic activity of this treatment arm was not significantly higher than rt-PA and US. Further, the lytic activity of OFP t-ELIP was similar to unencapsulated rt-PA. This outcome could be in part due to the low percentage of echogenic particles within OFP t-ELIP. However, combining OFP t-ELIP with US enhanced thrombolysis significantly compared to OFP t-ELIP alone. The results suggest that the use of Definity, US, and rt-PA could enhance thrombolysis substantially relative to treatment with unencapsulated rt-PA. Although lysis with OFP t-ELIP and US was not significantly higher than rt-PA alone, it approached the lytic efficacy achieved with rt-PA, Definity, and US. We demonstrated that OFP t-ELIP shielded rt-PA against degradation by PAI-1. We have previously reported that t-ELIP (albeit air-filled) can target clots^65^ and release the rt-PA dose upon Doppler US exposure^66^. Future studies should focus on the *in vivo* testing of rt-PA loaded in echogenic liposomes, to shield against PAI-1 degradation, in combination with ultrasound and an echocontrast agent such as Definity. Alternatively, further modification of OFP t-ELIP formulation or fabrication process^67^ could improve gas encapsulation and enhance the efficacy of sonothrombolysis.

We did not observe an improvement in thrombolytic efficacy with OFP t-ELIP and US relative to rt-PA combined with Definity and US, despite the ability of OFP t-ELIP to shield rt-PA against degradation by PAI-1. This apparent discrepancy may be explained by noting that the concentration of PAI-1 in the plasma used in this study was only 12.4 ng/ml. Although this concentration is in the physiological range for circulating blood^68^, PAI-1 concentrations *in vivo* are modulated by endothelial cells and platelets^69^. Moreover, stroke is commonly associated with prothrombotic comorbidities, such as hypertension, obesity and diabetes^70–73^, that cause dysregulation in PAI-1 activities and elevate plasma PAI-1 levels^74^. Others have shown that active PAI-1 levels increase more than a thousand-fold near thrombi *in vivo*^75^, which could adversely impact rt-PA treatment^74^. Accordingly, the half-life of rt-PA *in vivo* is only 3–6 min^76^. In addition, thrombi contain activated platelets that release PAI-1 and limit the local activity of rt-PA^77^. Furthermore, the PAI-1 concentration can be an order of magnitude higher in patients with platelet abnormalities^78^. The ability of OFP t-ELIP to encapsulate rt-PA and protect against degradation by PAI-1 suggests that this agent should be tested with clinically relevant PAI-1 levels.

The limitations of the *in vitro* model used in this study have been detailed previously^31–33^. Additionally, clots prepared from whole blood derived from healthy human donors may not encompass the diversity of clot composition and structure observed *in vivo*^46,48^. Platelets play an important role in the formation of thrombolysis-resistant clots *in vivo*^79–81^. Platelets are activated to a greater degree in arterial than in venous conditions. Although, in general, acute venous clots are easier to lyse than arterial clots, the coagulation cascade can be modulated to obtain retracted, rt-PA-resistant clots using venous blood. We have demonstrated the use of borosilicate glass to form clots that are more resistant to rt-PA thrombolysis than those prepared using soda lime or flint glass^82,83^. Under aqueous conditions, borosilicate glass acquires a negative surface charge^84^. Negatively-charged surfaces initiate the intrinsic pathway of the coagulation cascade *in vitro* by contact activation of Factor XII, which triggers clot formation^85,86^. *In vivo*, Factor XII is activated by contact with polyphosphates secreted by activated platelets in an arterial environment^86^. Although the mechanisms for activating Factor XII are different between *in vitro* and *in vivo* conditions, subsequent steps of the intrinsic pathway are similar, leading to the formation of clots^86^. Furthermore, we allow our clots to retract for 3 days, which makes them more resistant to rt-PA lysis^87^. Retraction modifies the structure and composition of clots substantially^88^. The serum from the clot is purged during retraction and the clot becomes more compact^89^. Retraction produces a tighter fibrin network clot permeability and the amount of soluble plasminogen, thereby increasing resistance to rt-PA^90,91^. Moreover, we characterized the fibrin content, porosity, and red blood cell content of the clots prepared using this protocol and showed that these values lie within the ranges reported for clots retrieved from stroke patients^82^.

The flow speed used in this study was fixed (0.3 cm/s), whereas a range of flow speeds (0 to 50 cm/s) has been reported clinically in ischemic middle cerebral arteries^92^. Nonetheless, slow flow represents a conservative scenario, as clot lysis and removal of degradation products has been reported to increase with flow^93^.

## Conclusions

We demonstrated that OFP t-ELIP are comprised of both liposomes that entrap microbubbles, along with other microbubbles that appeared visually similar to Definity. Lipid vesicles and rt-PA appeared to be co-localized, and OFP t-ELIP loaded with rt-PA protected as much as 76 ± 2% of enzymatic activity against degradation by PAI-1. Exposure to 220-kHz intermittent US in the presence of echocontrast agents enhanced the thrombolytic efficacy relative to either rt-PA or OFP t-ELIP alone. Additionally, sustained stable cavitation was nucleated by each contrast agent insonated at 220 kHz, despite the lack of large bubble formation.

## Methods

### OFP t-ELIP preparation

After preparation in Texas, OFP t-ELIP were packed on ice and shipped overnight to Cincinnati. The protocol for preparing OFP t-ELIP has been described in detail previously^94^. In brief, 1, 2-distearoyl-sn-glycero-3-phosphocholine (DSPC), 1,2-Dioleoyl-sn-glycero-3-phosphocholine (DOPC), 1,2-dipalmitoyl-sn-glycero-3-phosphoglycerol (DPPG) and cholesterol (Chol) were dissolved in a 46:23:23:8 molar ratio in chloroform and the solution evaporated overnight under vacuum. Subsequently, the film was rehydrated with 200 μl of rt-PA (1 mg/ml) and 60 μl of Mannitol (0.32 M) per mg of lipid. An ultracentrifuge (Model 5415D, Eppendorf, Hauppauge, NY, USA) was used at 13200 rpm for 20 min at room temperature (25 °C) to separate free rt-PA from the rt-PA associated with OFP t-ELIP. The liposomal pellet formed by centrifugation was re-suspended with 300 μL of 0.32 M Mannitol, and stored in glass vials (2-ml volume) for 2 h at −70 °C. The samples were lyophilized at −56 °C for 48 h and stored overnight at 4 °C in 2-ml glass vials with plastic screw caps and re-sealable silicone and PTFE septa. Using a similar protocol, FITC and rhodamine B-labeled OFP t-ELIP were prepared for microscopy studies. 1,2-dipalmitoyl-sn-glycero-3-phosphoethanolamine-N-(Lissamine Rhodamine B Sulfonyl) (Avanti Polar Lipids) labeled the lipid in the echogenic liposomes as red in the fluorescence images. Commercially-available FITC-labeled rt-PA (Abcam, Cambridge, MA, USA) was used in this study. The green color associated with FITC served as a surrogate for the presence of rt-PA in the fluorescence images. The lipids 1,2-distearoyl-sn-glycero-3-phosphocholine (DSPC), 1,2-Dioleoyl-sn-glycero-3-phosphocholine (DOPC), and 1,2-dipalmitoyl-sn-glycero-3-phosphoglycerol (DPPG) (Avanti Polar Lipids) and Cholesterol (Chol) (Sigma-Aldrich, St. Louis, MO, USA) were mixed in a 39:23:23:15 molar ratio (total lipid mass 2.5 mg), and 2% (v/v) of 1 mg/ml 1,2-dipalmitoyl-sn-glycero-3-phosphoethanolamine-N-(Lissamine Rhodamine B Sulfonyl) (Avanti Polar Lipids) was added into the mixture. The chloroform was evaporated under argon and vacuumed overnight. The dried lipid film was hydrated with 75 μg FITC-rtPA (1 μg/ul) and 175 μl of 0.32 M mannitol. The sample was frozen at −80 °C for at least 1 hour, after which the frozen mixture was thawed at room temperature. The FITC-tPA-ELIP were separated from free FITC-rt-PA by centrifuging at 13,200 rpm for 20 min. The liposomal pellet was re-suspended with 200 μl of 0.32 M mannitol, and frozen at −80 °C for 2 hours. Subsequently the mixture was lyophilized at −56 °C for 48 h and stored at 4 °C in a 2 ml-glass vial.

The prepared vials of OFP t-ELIP or FITC- and rhodamine B-labeled OFP t-ELIP contained 4 mg of lipid. Prior to reconstitution with OFP, the vials were evacuated for 5 min using a laboratory wall vacuum at 10 mm Hg and reloaded with 2 ml of OFP through the septum with a 27-gauge needle. The OFP t-ELIP vials were reconstituted three hours after adding OFP to the vial headspace. Specifically, a 22-gauge needle was used to vent the vial, and the OFP t-ELIP were reconstituted with 0.4 mL of deionized, air saturated (100 ± 2%), and 0.2 μm filtered water, unless stated otherwise. Subsequently, the OFP t-ELIP were diluted in hFFP to achieve an rt-PA concentration of 3.15 μg/ml and used in thrombolysis experiments.

### Spectrophotometric measurement of rt-PA enzymatic activity

The concentration of enzymatically active rt-PA associated with OFP t-ELIP was assessed using a chemical substrate (Chromogenix S-2288, DiaPharma Group, Inc., Westchester, OH, USA) that produces a yellow chromophore when exposed to rt-PA^23,31^. A standard curve was created with commercially available rt-PA (Activase, Genetch, San Francisco, CA, USA). Reconstituted OFP t-ELIP was diluted into a solution of 0.5% phosphate buffer solution (PBS) (Sigma-Aldrich, St. Louis, MO, USA) and 0.01% (v/v) Triton X-100 (Sigma-Aldrich, St. Louis, MO, USA) in 96-well plates. Spectrophotometric measurement was performed using a plate reader (Cytation 5, BioTek Instruments, Winnoski, VT, USA) by measuring the change in absorbance of the solution at 405 nm over 5 min. The loading ratio of OFP t-ELIP was calculated. The loading ratio represents the total rt-PA associated with OFP t-ELIP relative to the total amount of rt-PA added during OFP t-ELIP manufacturing. The loading ratio (LR) was defined as:

1 $$LR=\frac{{A}_{1}\,}{{A}_{total}}\times 100 \% ,$$

where *A*_*total*_ depicts the enzymatic activity of the rt-PA added during OFP t-ELIP fabrication (300 μg/ml), and *A*_1_ represents the enzymatic activity of the rt-PA obtained after treating OFP t-ELIP with 1% Triton X-100. This concentration of Triton X-100 disrupts the lipid bilayer and releases the encapsulated rt-PA^23^.

### Preparation and testing of plasma

Eight units of human fresh-frozen plasma (hFFP) were purchased from the Hoxworth Blood Center (Cincinnati, OH, USA), thawed, pooled together, and stored at −80 °C as 50-mL aliquots. An ELISA kit (BMS 2033, ThermoFisher Scientific, Waltham, MA, USA) was used to confirm that the frozen and thawed hFFP contained physiological concentrations of plasminogen activator inhibitor-1 (PAI-1). The concentration of PAI-I in hFFP was 12.4 ± 0.5 ng/mL, which is within the physiological range^68^. Before use in experiments, hFFP was thawed and allowed to reach atmospheric gas equilibrium at 37 °C in a 500 ml beaker for 2 h.

### Evaluation of rt-PA inhibition by PAI-1

Cell culture plates (Fisher Scientific, 96 wells) were coated with 50 μg/ml of human fibrinogen (Fgn, Calbiochem) in 80 μl PBS overnight at 4 °C. On the following day, the well contents were aspirated and washed three times with PBS-T0.05% (0.02 M phosphate-buffered saline, pH 7.4, with 0.05% Tween 20). Subsequently, the fibrinogen was converted to fibrin by incubating with 1 U/ml of thrombin and 0.56 U/ml of aprotinin in 80 μl of PBS for 20 min at 37 °C. After the incubation, the wells were washed three times with PBS-T0.05%. Free rt-PA and OFP t-ELIP were diluted to an rt-PA concentration of 5 μg/ml, and incubated in the fibrin-coated wells with 80 μl of 5 μg/ml rt-PA and OFP-tELIP overnight at 4 °C. On the next day, the wells were washed three times with PBS-T0.05% and incubated in the wells with 80 μl of 500 ng/ml (or 40 ng) Plasminogen Activator Inhibitor-1 (PAI-1, 528205–50 μg, Calbiochem, Billerica, MA, USA) for 15 min at 37 °C to inactive rt-PA activity. After incubation, the wells were washed three times with PBS-T0.05%, and 80 μl of PBS-0.5%Triton was added to each well and allowed to sit at room temperature for 10 min. Subsequently, 20 μl of chromogenic substrate (Chromogenix S-2288, DiaPharma Group, Inc., Westchester, OH, USA) was added to the lysate and incubated for 15 min at 37 °C. The optical absorbance of each well was measured at 405 nm using a microplate reader (SYNERGY/H1, BioTek instruments, Inc., Winooski, Vermont, USA) to test rt-PA activity.

### Microscopic imaging of OFP t-ELIP

#### Differential interference contrast microscopy

To assess the morphology of OFP t-ELIP and Definity, we performed differential interference contrast (DIC) microscopy. Either Definity or OFP t-ELIP (10 μL, undiluted) was placed on a plasma-treated polystyrene microscope slide (Ted Pella, Redding, CA, USA), covered with a glass coverslip and visualized with a Axioplan 2 Imaging microscope (Zeiss, Thornwood, NY, USA) with DIC imaging functionality. This microscope was equipped with a 63x oil immersion objective (Plan Apochromat, Zeiss, Thornwood, NY, USA) that had a numerical aperture of 1.4. A charged coupled device camera (Axiocam MRM, Zeiss, Thornwood, NY, USA) was used to acquire either still images or videos at a frame rate of 15 frames/second. Images were also acquired with OFP t-ELIP reconstituted in undersaturated (dissolved gas saturation = 80 ± 2%), deionized, and 200 nm-filtered water. This protocol was used to eliminate gas from OFP t-ELIP, to enable visualization of the liposome morphology. Subsequent images were acquired with OFP t-ELIP reconstituted using air-saturated (dissolved gas saturation = 100 ± 2%) water to retain echogenicity. Images were acquired with the microscope focused near the coverslip (top) to visualize microbubbles as well as near the polystyrene slide (bottom) to visualize liposomes. To assess the location of rt-PA in OFP t-ELIP, fluorescence microscopy was performed with the same system and a 100x oil-immersion objective (Plan Apochromat, Zeiss, Thornwood, NY, USA). For these measurements, OFP t-ELIP were diluted 100-fold in air-saturated water (dissolved gas saturation = 100 ± 2%) before imaging with fluorescence microscopy. Chroma filter sets 41001 and 41002b (Chroma Technology Corporation, Bellows Falls, VT, USA) were employed to visualize the green and red florescence associated with FITC and rhodamine B, respectively. The images obtained were superimposed with DIC images.

### Attenuation and size distribution measurements

The attenuation and size distribution of Definity have been reported previously^40,95,96^. The size distribution of OFP t-ELIP was measured a Coulter counter (Multisizer 4, Beckman Coulter, Brea, CA, USA). Briefly, OFP t-ELIP were diluted (1:20,000 v/v) in air-saturated PBS at room temperature for the size distribution measurements. The size distributions were scaled to a dilution of 1:600, consistent with the attenuation measurements described below. The attenuation spectrum of OFP t-ELIP was measured in the frequency range of 2–25 MHz as described previously^40^. Attenuation spectra were acquired at a dilution of (1:600) and peak negative pressure of 31 kPa.

### Human whole-blood clot preparation

The protocol and experimental apparatus used for *in vivo* thrombolysis studies have been described in detail elsewhere^23,31–33^. Briefly, venous blood was drawn from four healthy donors following a protocol approved by the Institutional Review Board at the University of Cincinnati (IRB protocol number 2012–2575). Written informed consent was obtained from all donors, and all research was performed in accordance with relevant guidelines and regulations. Clots adherent to 7–0 silk sutures were created using borosilicate glass capillaries (1.12 mm inner diameter, World Precision Instruments, Inc., Sarasota, FL, USA) in accordance with previously reported protocols^31,32^. Each clot was incubated at 37 °C for 3 hours. The clots were placed in disposable culture tubes and allowed to retract at 4 °C for a minimum of 3 days^97^. This clot model produces retracted clots that are fibrin and erythrocyte rich, and demonstrate reduced susceptibility to rt-PA thrombolysis^33^. The clots were used within 14 days after an initial 3 days of retraction. Previous studies from our group have demonstrated that the lytic susceptibility to rt-PA does not vary among clots stored for up to 14 days^50^.

### Time-lapse microscopy thrombolysis *in vitro*

**Figure 7:**
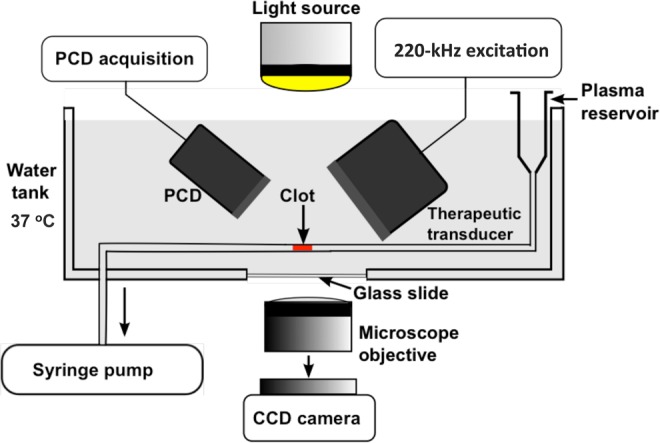
A schematic of the experimental set-up used in the *in vitro* flow system. CCD = Charged Coupled Device, PCD = Passive Cavitation Detector.

For *in vitro* thrombolysis studies, the clots were mounted in a flow system (Fig. 7) equipped with a time-lapse microscope (IX71, Olympus Corporation, Center Valley, PA, USA). A 1200 × 900 μm area encompassing the clot was visualized during the treatment period with a CCD camera (Retiga-2000R, Q Imaging, Surrey, BC, Canada) with a 7.4 μm pixel resolution and a frame rate of 2.33 Hz. A custom-designed transducer (220 kHz center frequency, 38 mm aperture diameter) fabricated at Nanjing University was used to insonate the clot and the perfusate within the flow system. A function generator (33250A, Agilent Technologies, Inc., Santa Clara, CA, USA) was used to generate radio frequency (RF) signals, which were boosted using a power amplifier (Model 75A250, Amplifier Research, Souderton, PA, USA) and relayed to the transducer through a custom-built impedance matching network. An intermittent US insonation scheme (50 s on, 30 s off) was employed (220 kHz frequency, and 0.44 MPa peak-to-peak pressures) for the treatment duration of 30 min^32^.

The acoustic field and the *in situ* acoustic pressure along the clot were assessed using a calibrated broadband hydrophone (0.5 mm aperture, TC 4038, Teledyne Reson Inc. Goleta, CA, USA). Cavitation signals were monitored using a passive cavitation detector (19 mm aperture, 2.25 MHz center frequency, 595516 C, Picker Roentgen GmbH, Espelkamp, Germany). The received RF signal was filtered with a 10 MHz low-pass filter (J73E, TTE Inc, Los Angeles, CA, USA) and amplified using a wideband low-noise amplifier (CLC100, Cadeka Microcircuits LLC, Colorado, USA). The signal was digitized (12-bit resolution, 10 ms duration, 31.25 MHz sampling frequency) using a digital oscilloscope (Picoscope 4227, PicoTech, St. Neots, Cambridgeshire, UK). Image analysis was used to assess the fractional clot loss in response to (1) plasma alone, (2) rt-PA, (3) OFP t-ELIP, (4) rt-PA and US, (5) OFP t-ELIP and US, (6) Definity and US, or (7) rt-PA, Definity, and US. A total of 112 clots were tested in this study (n = 16 clots per group). OFP t-ELIP were used at a particle concentration of 1.3 × 10^8^ particles/mL, which resulted in an rt-PA dose of 3.15 μg/mL, consistent with steady state intravenous dose of rt-PA. A constant volume flow rate of 0.65 mL/min (flow velocity of 0.3 cm/s) was maintained using a syringe pump (Model 44, Harvard Apparatus Co. Inc., South Natick, MA, USA) in withdrawal mode. This flow rate is within the flow rates reported in occluded middle cerebral arteries^92^. The concentration of Definity was 2.4 × 10^7^ microbubbles/mL, consistent with our previous work^23,33^.

The average clot width was computed over the field of view and corrected for suture size (64 ± 9 μm). The fractional clot loss (FCL) and average lytic rate (ALR) were used to quantify the efficacy of thrombolysis as reported previously^31^. For clots that lysed completely before 30 min, the ALR was computed using the time required to achieve 100% FCL. If the clot completely separated from the suture during treatment, the ALR was calculated based on the time point and FCL just before separation. The power spectrum of filtered RF signals was computed offline using MATLAB (The Mathworks, Natick, MA, USA), and stable cavitation emissions quantified by integrating the power in ultraharmonic (UH) bands between 220 kHz and 2 MHz over a 2 kHz band centered around each UH frequency.

### Statistical analysis

The D’Agostino’s-Pearson test was used to test the normality of the data obtained from experiments. The data was found to be non-normal. Accordingly, the lytic efficacy of the different treatment protocols was compared using a nonparametric Kruskal-Wallis test. *Post-hoc* analysis was performed using Dunn’s non-parametric pairwise multiple comparison test to assess whether the differences in thrombolysis between the treatment groups were statistically significant relative to rt-PA alone. The lytic efficacy of OFP t-ELIP alone and OFP T-ELIP combined with US were compared using the Mann-Whitney test. The ultraharmonic cavitation dose was compared using the Kruskal-Wallis test. Statistical analysis was performed using GraphPad Prism 7 (GraphPad Software Inc., La Jolla, CA, USA). A *p*-value of less than 0.05 was considered statistically significant.

## Supplementary information

Supplementary Video 1

Supplementary Video 2

## Data Availability

The data generated during and/or analyzed during the current study are available from the authors upon reasonable request.
